# Pulmonary Hypertension as a Rare Complication After Orthotopic Liver Transplant in a Patient With Non-alcoholic Steatohepatitis (NASH) Cirrhosis Complicated by Hepatopulmonary Syndrome

**DOI:** 10.7759/cureus.24740

**Published:** 2022-05-04

**Authors:** Apaar Dadlani, Michael Eiswerth, Armando Bosch, Tyler Sharpe

**Affiliations:** 1 Internal Medicine, University of Louisville School of Medicine, Louisville, USA

**Keywords:** cirrhosis, intrapulmonary shunt, liver transplant, pulmonary hypertension, hepatopulmonary syndrome

## Abstract

Hepatopulmonary syndrome is a phenomenon that results in an intrapulmonary shunt leading to dyspnea and hypoxemia with poor response to oxygen supplementation. It is now an indication for liver transplantation; however, some transplants result in subsequent pulmonary hypertension. Postulated mechanisms include unmasking of underlying pulmonary hypertension with liver transplant and increased pulmonary vascular resistance due to increased blood flow in hepatopulmonary syndrome. In this case, we describe pulmonary hypertension developing after orthotopic liver transplant in a cirrhotic patient with hepatopulmonary syndrome.

## Introduction

Hepatopulmonary syndrome (HPS) is a phenomenon that results in a right to left intrapulmonary shunt leading to dyspnea and hypoxemia with poor response to oxygen supplementation. HPS became an indication for liver transplants after evidence showed a dismal survival rate in patients not receiving transplants [1]. In this case, we describe a rare occurrence of pulmonary hypertension developing after orthotopic liver transplant (OLT) in a cirrhotic patient with HPS.

This case was accepted for poster presentation at the American College of Gastroenterology (ACG) 2021 conference in October 2021.

## Case presentation

A 61-year-old female with a past medical history of non-alcoholic steatohepatitis (NASH) cirrhosis decompensated with esophageal varices and hepatic encephalopathy underwent a liver transplant for HPS. She was treated symptomatically with supplemental oxygen and garlic before a successful OLT could be performed. After the transplant, she reported subjective improvement in energy level and was using varying levels of oxygen supplementation depending on her level of activity.

**Figure 1:**
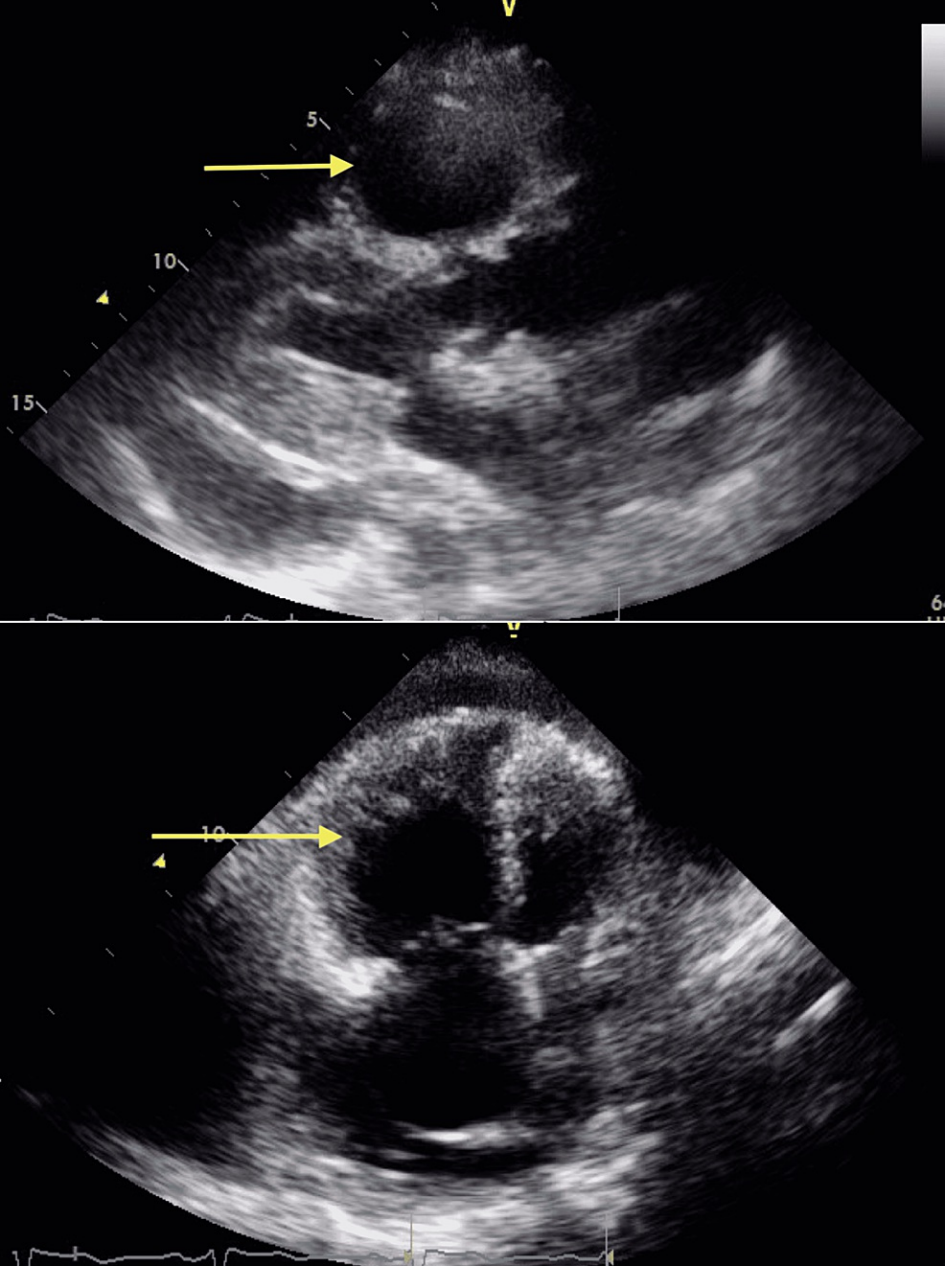
Echocardiogram showing moderate right ventricular dilation (arrows) with leftward diastolic displacement of the interventricular septum

Two years after the transplant, she presented with progressive dyspnea on exertion. Chest x-ray, CT chest with contrast and lower extremity dopplers did not reveal any acute pathology. An echocardiogram showed an ejection fraction of 65%, moderate right ventricular dilation with a leftward diastolic displacement of the interventricular septum (Figure 1); however, the right ventricular systolic function was preserved. No left ventricular or any valvular abnormality was noted. Subsequent left heart catheterization was negative for evidence of coronary disease. Right heart catheterization (RHC) revealed elevated mean pulmonary artery pressure (mPAP) of 66 mm Hg (normal 14 ± 3 mm Hg). Left ventricular end-diastolic pressure was 16 mm Hg and right atrial pressure was 17 mm Hg. Right ventricular end-diastolic pressure was 112 mm Hg, consistent with severe pulmonary arterial hypertension (PAH). Her cardiac output (CO) was calculated to be 2.17 L/min (normal 4-8 L/min). Upon arrival to our facility, she required 2 L/min O_2_ via nasal cannula and was started on intravenous epoprostenol and riociguat for severe PAH.

Repeat RHC during hospitalization showed mild improvement of mPAP to 57 mm Hg and CO to 4 L/min with the above treatment. The patient’s hospital course was complicated by worsening shortness of breath ultimately requiring intubation. She also developed encephalopathy, acute kidney injury, and acute blood loss anemia from variceal bleed. Unfortunately, the patient’s condition continued to deteriorate during the hospitalization, and comfort measures were initiated.

## Discussion

HPS and porto-pulmonary hypertension (POPH) are the two main pulmonary complications that result due to advanced cirrhosis. HPS was first described in the literature in 1977 when cirrhotic patients were found to have exercise-induced hypoxemia [2]. It was hypothesized to be from an intrapulmonary shunt similar to the pathophysiology of hepatorenal syndrome. Generally, patients with HPS are asymptomatic and the most frequent symptom is progressive dyspnea. Interestingly, platypnea and orthodeoxia are seen in these patients due to preferential vasodilation of the basal part of the lungs [3]. The pulmonary vascular dilation creates an intrapulmonary shunt causing hypoxemia. The diagnostic triad of HPS consists of arterial deoxygenation (PaO_2_ < 80 mm Hg), evidence of intrapulmonary vascular dilation (IVPD), and evidence of liver disease or portal hypertension. IVPD is often demonstrated by contrast-enhanced echocardiography or perfusion lung scans [4]. It is estimated that 5%-23% of cirrhotic patients develop HPS [1].

The primary insult of IVPD is thought to occur secondary to the overproduction of pulmonary nitric oxide (NO), as evidenced by the elevated levels of NO in HPS patients that subsequently resolve after OLT [4]. However, the mechanism of this theory is poorly understood. One proposed mechanism involves bacterial translocation from impaired endotoxin clearance by the liver, causing macrophage recruitment to pulmonary vessels resulting in inflammation and nitric oxide production [3]. Our patient was initially treated with garlic, which is thought to inhibit NO synthesis in macrophages [5].

Treatment of HPS primarily supportive with supplemental oxygen. The severity of HPS is poorly correlated with the extent of liver disease measured by the model for end-stage liver disease (MELD) or Childs-Pugh scores [1]. Therefore, it is now an indication for liver transplantation, and the majority of transplanted patients achieve complete resolution of their HPS. Patients with moderate to severe HPS (PaO_2_ < 60 mm Hg) receive “MELD score exceptions” for their transplant eligibility [6]. The five-year-survival in patients with HPS without treatment is estimated to be 23% [3]. However, as demonstrated in our case, some liver transplant recipients may subsequently develop pulmonary hypertension.

The first documented case of pulmonary hypertension developing after liver transplant was in 1992, two and a half years liver transplantation [7]. This patient did not have prior HPS and post-mortem autopsy revealed intimal and medial hyperplasia of the pulmonary vasculature without evidence of pulmonary emboli. Since this publication, there have been approximately 14 documented cases in the literature of this phenomenon, including five pediatric patients [4,7-15]. Authors have postulated two main mechanisms by which this phenomenon occurs. The first mechanism is that the resolution of HPS (intrapulmonary vasodilation) after liver transplant unmasks underlying pulmonary hypertension. The second leading mechanism is that the increased blood flow in HPS results in remodeling of pulmonary arterioles, which subsequently increases pulmonary vascular resistance, similar to the mechanism in a congenital left-to-right intracardiac shunt [12]. Zopey et al. described a case series in which three patients achieved spontaneous resolution of HPS and subsequently developed POPH before liver transplantation [13]. This highlights the intricate balance between pulmonary vasodilators and vasoconstrictors. The interplay and dysregulation of signaling pathways in the pulmonary vasculature that leads to HPS and POPH could be more closely related than previously thought.

## Conclusions

Our case adds to the growing recognition of the development of pulmonary hypertension after liver transplant in patients with HPS. It is important for providers to be aware of this phenomenon, as it carries a high mortality. Further research regarding this condition and the interplay between pulmonary vascular constriction and dilation in patients with liver disease is needed to improve outcomes and prevent cases such as ours from transpiring.
